# Global mapping transcriptional start sites revealed both transcriptional and post-transcriptional regulation of cold adaptation in the methanogenic archaeon *Methanolobus psychrophilus*

**DOI:** 10.1038/srep09209

**Published:** 2015-03-18

**Authors:** Jie Li, Lei Qi, Yang Guo, Lei Yue, Yanping Li, Weizhen Ge, Jun Wu, Wenyuan Shi, Xiuzhu Dong

**Affiliations:** 1 State Key Laboratory of Microbial Resources, Institute of Microbiology, Chinese Academy of Sciences, No.1 Beichen West Road, Beijing 100101, People's Republic of China; 2 Novogene Bioinformatics Institute, 21st Floor, Jinma building B area, Xueqing Road, Beijing 100083, People's Republic of China; 3 Department of Microbiology, Immunology and Molecular Genetics, University of California, 10833 Le Conte Avenue, Los Angeles, CA90095, USA

## Abstract

Psychrophilic methanogenic Archaea contribute significantly to global methane emissions, but archaeal cold adaptation mechanisms remain poorly understood. Hinted by that mRNA architecture determined secondary structure respond to cold more promptly than proteins, differential RNA-seq was used in this work to examine the genome-wide transcription start sites (TSSs) of the psychrophilic methanogen *Methanolobus psychrophilus* R15 and its response to cold. Unlike most prokaryotic mRNAs with short 5′ untranslated regions (5′ UTR, median lengths of 20–40 nt), 51% mRNAs of this methanogen have large 5′ UTR (>50 nt). For 24% of the mRNAs, the 5′ UTR is >150 nt. This implies that post-transcriptional regulation may be significance in the psychrophile. Remarkably, 219 (14%) genes possessed multiple gene TSSs (gTSSs), and 84 genes exhibited temperature-regulated gTSS selection to express alternative 5′ UTR. Primer extension studies confirmed the temperature-dependent TSS selection and a stem-loop masking of ribosome binding sites was predicted from the longer 5′ UTRs, suggesting alternative 5′ UTRs-mediated translation regulation in the cold adaptation as well. In addition, 195 small RNAs (sRNAs) were detected, and Northern blots confirmed that many sRNAs were induced by cold. Thus, this study revealed an integrated transcriptional and post-transcriptional regulation for cold adaptation in a psychrophilic methanogen.

Cold-adaptive methanogenic Archaea (methanoarchaea) contribute significantly not only to methane emissions from the wetlands in cold regions of the Earth, but also to the low-temperature biogas fermentations^1^. However, the mechanisms of cold adaptation used by these psychrophilic Archaea have not been well studied except for the Antarctic *Methanococcoides burtonii*^2^,^3^,^4^, probably because only a few cold-adaptive Archaea have been cultured. In this study, a domesticated psychrophilic methanogen *Methanolobus psychrophilus* R15 isolated from the cold Zoige wetland on Tibetan Plateau^5^, was used to explore archaeal cold adaptation mechanisms. Previous transcriptomic analysis of R15 indicated that expression of 40% of its genes responded to changes in temperature and that the genes specifically related to RNA degradation were up-regulated in response to cold^6^, implying that mRNA turnover may play a role in the cold-responsive gene regulation.

Cold has various impacts on biological systems, such as increasing the rigidity of the cell membrane and stabilizing mRNA secondary structure. The latter affects mRNA turnover and translation^7^. Because mRNA secondary structure responds to cold more promptly than proteins^7^, changes in mRNA secondary structure may be a preferred mode in cold-responsive regulation. For example, the cold shock protein gene *cspA* of *E. coli* encodes an RNA chaperone and transcribes a large 5′ untranslated region (UTR) of mRNA which determines the transcript's cold stability and translational conformation in the cold^8^. This unique architecture of the *cspA* mRNA allows it to function as a RNA thermometer^9^. Our recent work demonstrated that the mRNAs for *mtaA* and *mtaC*, which encode the methylcobalamin:coenzyme M methyltransferase and methanol corrinoid proteins that are the key enzymes in methanol-derived methanogenesis of a cold-adaptive methanogen, all have long 5′ UTRs, that contribute to the transcripts' stability at low temperatures^10^.

Precise transcript architecture can reveal diverse *cis*-RNA elements, including the 5′ and 3′ UTR and non-coding RNAs, those contribute significantly to gene regulation in Eukaryotes^11^,^12^. However, whole-transcript RNA sequencing often fails to identify the precise transcriptional architectures due to under-representation of the 5′ and 3′ ends of transcripts. By using differential RNA-seq (dRNA-seq), an approach that discriminates the primary (5′ tri-phosphorylated, 5′ PPP) from the processed (5′ mono-phosphorylated, 5′ P) transcripts, Jäger *et al*. have identified the genome-wide transcription start sites (TSSs) for *Methanosarcina mazei* Gö1. They found that the majority of the mRNAs possess long 5′ UTRs and discovered more than two hundreds of sRNAs^13^, suggesting the importance of post-transcriptional regulation in methanoarchaea. Following that study, this newly developed dRNA-seq approach has also been used in human pathogens *Helicobacter pylori*^14^, *Enterococcus faecalis*^15^, and cyanobacteria *Synechocystis* sp. PCC6803 and *Anabaena* sp. PCC7120^16^,^17^. These studies have identified genome-wide TSSs and revealed the response of the primary transcriptomes of bacteria to the environment.

Based on the hypothesis that mRNA structure-dependent post-transcriptional regulation could play a role in cold-adaptive methanogens, this work was designed to reveal the detailed changes of the genome-wide transcriptional architecture of *M. psychrophilus* R15 in response to cold. dRNA-seq was used to generate a temperature-dependent genome-wide TSS atlas of *M. psychrophilus* R15. The TSSs were further refined by assembly of the 5′-end libraries with the whole-transcript libraries, and some TSSs were experimentally confirmed. Extensive analyses were performed to understand the dynamic transcriptome in connection with the cold-response of this psychrophilic archaeon.

## Methods

### Strain cultivation and RNA extraction

*M. psychrophilus* R15 was grown at 8 and 18°C in a mineral medium containing 20 mM trimethylamine under gas phase of 80:20 N_2_:CO_2_ as described^5^. Cells were harvested from the mid-log phase culture at 4°C, and total RNA was extracted using TRIzol. Briefly, frozen pellets collected from 20 mL of culture were lysed in 1 mL of TRIzol Reagent (Ambion). After 5 min of incubation at room temperature, 200 μL of chloroform was added. The suspension was shaken vigorously and centrifuged at 12,000 *g* for 15 min. The supernatent was combined with 500 μL of isopropanol and centrifuged at 12,000 *g* for 10 min. The pellet was washed with 70% ethanol, air-dried, and dissolved in RNase-free water. RNA purity and integrity were assessed using a NanoPhotometer spectrophotometer (Implen) and RNA Nano 6000 Assay Kit of the Agilent Bioanalyzer 2100 system (Agilent Technologies), respectively.

### Construction of 5′-end cDNA libraries and whole-transcript sequencing cDNA libraries

To obtain reliable gene expression data, external synthetic spike-in RNA controls were included to evaluate the sensitivity and accuracy of the RNA-sequencing experiments as described by Loven *et al*^18^. After total RNA extraction, External RNA Controls Consortium (ERCC) RNA Spike-In Control Mixes (Ambion) were added to each sample as an internal standard according to manufacturer's instructions. Using 5 μg of the total RNA plus spike-in control, 5′-end cDNA libraries for dRNA-seq were constructed as described by Sharma *et al*^14^ with some minor modifications. Briefly, rRNA was depleted at a high efficiency (Supplementary Table S1) using Ribo-Zero™ rRNA Removal Kit (Epicentre). To discriminate the primary transcripts from those with processed 5′ ends, two cDNA libraries were constructed as follows: a (+) library was enriched in primary transcripts (5′ tri-phosphated, 5′ PPP) by using the Terminator™ 5′-phosphate-dependent exonuclease (TEX, Epicentre) to deplete the processed RNAs (5′ mono-phosphated, 5′ P). A (−) library without TEX treatment that contained both 5′ PPP- and 5′ P-transcripts was also prepared. Both the TEX-treated and untreated RNA samples were then subject to a treatment of tobacco acid pyrophosphatase (TAP, Epicentre) to generate 5′ P-transcripts for linker ligation. Next, using an RNA-Seq Library Preparation Kit for Transcriptome Discovery (Gnomegen), dRNA-seq cDNA libraries were constructed in accordance with the manufacturer's recommendations. The cDNAs were size-fractioned within the range of 300 to 600 nt on agarose gels and purified using a QIAquick Gel Extraction Kit (Qiagen). In addition, for TSS refinement and verificaion, standard strand-specific whole-transcript sequencing cDNA libraries (w library) were constructed for the total RNAs using NEBNext® Ultra™ Directional RNA Library Prep Kit for Illumina® (New England Biolabs) according to the manufacturer's instructions.

### High-throughput sequencing and quality control (QC)

cDNA libraries were sequenced on an Illumina HiSeq 2000 platform. The short reads sequenced from one end of the cDNA fragments (single-end sequencing) was used for dRNA-seq libraries, but paired-end sequencing was used for the whole-transcript libraries; both produced reads of 100 bp in length. Images generated by a sequencing machine were converted to raw sequencing data using base calling (CASAVA version 1.8.2). Adapter sequences were trimmed from reads and then subjected to the following pre-processed QC steps to discard all of the reads that meet any of the following thresholds: truncated reads of ≤1/3 of the original reads in length; reads with ≥10% uncalled bases (Ns); and reads with ≥50% of low-quality bases (PHRED quality scores ≤5).

### Read mapping and gene quantification

QC filtered reads were aligned to the *M. psychrophilus* R15 reference genome (ftp://ftp.ncbi.nlm.nih.gov/genomes/Bacteria/Methanolobus_psychrophilus_R15_uid177925/) and ERCC spike-in sequences (https://www.lifetechnologies.com/order/catalog/product/4456740) using Bowtie (version: 0.12.7). Sequence alignment was performed with default parameters except for allowing three mismatches^19^. For all the comparisons, read counts were normalized to the reads per kilobase of genic (or genomic) region per million mapped reads (RPKM) to obtain the relative levels of expression^20^.

### TSS calling and statistics

Mapped reads were clustered with at least one base overlapping the construct reads group, *contig*. The 5′ end(or the read ‘head’)of each clustered contigs was counted in the whole genomic region. The site with the highest 5′ end reads (no fewer than 10) of a contig was considered as the primary transcription start site (TSS) of a gene; other determined TSSs with fewer 5′ end reads (but not fewer than 5) than the primary TSS in same gene were assigned as secondary TSSs. All the gene TSSs (gTSS) obtained from sequencing reads were refined with intensive manual correction by reference to the whole-transcript sequencing data following the rule that a continuous coverage of whole-transcript reads is sequenced from a TSS to its downstream start codon but 20–30 nt missing at the 5′ terminal is allowed. To compare the differential expressions among dRNA-seq libraries generated from the 8°C- and 18°C-cultures, 5′ end read (head read) numbers per million mapped reads (HRPM) were defined for each TSS by normalizing the total head reads in 50 nt-length downstream the TSS to the total mapped million reads in each library. All used scripts were written in Python and available on request. For each category, TSS at two temperatures (8 and 18°C) samples were merged and used to detect promoter motifs. MEME with default parameter was used to search for sequence motif features within 50 nt upstream of predicted TSSs^21^. Motif sequence logos were created using WebLogo (http://weblogo.threeplusone.com/) developed by Schneider and Stephens^22^,^23^.

### Operon prediction

DOOR 2.0^24^ and Rockhopper^25^ were used to predict operons. Operon maps generated by integrating the two predictions were confirmed manually and refined by the criteria that each operon has a continuous coverage by the whole-transcript sequencing reads as well as a mapped upstream gTSS.

### 5′-RACE

To confirm the dRNA-seq data, TSSs of transcripts with different-sized 5′ UTRs were confirmed using 5′-RACE following published protocols^26^,^27^,^28^. 20 μg of the total RNA was digested with 25 units of tobacco acid pyrophosphatase (TAP, Epicentre) at 37°C for 2 h, an aliquot of RNA extract without TAP treatment was included as control. The TAP-treated and control RNAs were precipitated using isopropanol and dissolved in RNase-free water. Then they were ligated to 500 pmol 5′ RNA adapter (5′-CAGACUGGAUCCGUCGUC-3′; Integrated DNA Technologies) at their 5′ ends by 50 units of T4 RNA ligase (Ambion) at 17°C for 12 h. Adaptor-ligated RNA was precipitated again with isopropanol, and 2 μg served as the template for reverse-transcription (RT). Gene-specific primers (RT-primer in Supplementary Table S2) that targeted the region less than 200 nt downstream of the start codons were used for reverse transcription using 200 units of SuperScript III reverse transcriptase (Invitrogen). All the enzymatic procedures were performed in the presence of 20 units of RNasin Ribonuclease Inhibitors (Promega). Then a 2 μL aliquot of the RT reaction was used for the first-run PCR amplification with 20 pmol of each RT-primer and adapter-specific primer; and 1 μL aliquot of products served as the template of the second round PCR using 20 pmol adapter-specific and a gene-specific nested primer (R primer in Supplementary Table S2) upstream of the RT-primer. PCR products were separated on 2.5% high resolution agarose gel, and bands of interest were excised, gel-eluted (QIAquick; Qiagen), and cloned into pMD19-T vector (TaKaRa). After transformation into *E. coli* DH5α, plasmid inserts were screened using colony PCR. The PCR fragments were then purified and sequenced using an ABI 3730xl DNA Analyzer (Applied Biosystems).

### Primer extension assay

A primer extension assay was used to quantify the levels of expression of different gTSSs used by a single gene. Briefly, primer (PE-RT in Supplementary Table S2) was [γ-^32^P] GTP labelled at the 5′ end by T4 polynucleotide kinase. It was used for both DNA sequencing and primer extension reactions, as described previously^29^,^30^. For the primer extension reactions, 10 μg of total RNAs and 10 pmol of [γ-^32^P] GTP-labelled primer were mixed, denatured at 65°C for 10 min, and chilled on ice immediately. Then, 4.5 μL of 5× reverse transcription buffer (Invitrogen), 1 μL of 0.1 M DTT, 1 μL of 10 mM dNTP, 1 μL RNasin Ribonuclease inhibitor (Promega), and 1 μL of Superscript III reverse transcriptase (200 U/μL, Invitrogen) were added to the RNA/primer mixture and incubated at 55°C for 1 h. The reaction was terminated by incubation at 70°C for 15 min. Then 50 μL RNase reaction mix (100 mg/mL salmon sperm DNA, 20 μg/mL RNase A) was added. After incubation at 37°C for 15 min, the reaction products were extracted with phenol and chloroform and precipitated with ethanol and glycogen overnight at −80°C. DNA sequencing reactions were performed with the same [γ-^32^P] GTP-labelled primer by using a Sequi Thermo EXCEL II DNA sequencing system (Epicentre) according to the manufacturer's instructions. The products of the primer extension and DNA sequencing reactions were resuspended separately in the same sequencing loading buffer, denatured at 90°C for 5 min, and run on a 6% acrylamide sequencing gel. After electrophoresis, the gels were subjected to autoradiography on X-ray film.

### Northern blot analysis

2–5 μg total RNA per lane was denatured for 10 min at 65°C in the loading buffer containing 95% (v/v) formamide and separated on 8% polyacrylamide gels containing 7.6 M urea with a low range ssRNA ladder (New England Biolabs). After separation, RNAs were transferred onto Hybond-N+ membranes (GE Healthcare) by electroblotting and cross-linked to the membrane using UV. Membranes were prehybridized at 42°C, followed by hybridization with 10 pmol 5′-biotin-labeled DNA probes (Supplementary Table S2) for 12 h. After three rounds of washing for 10 min each in 1×, 0.2×, and 0.1× SSC–0.1% SDS solutions, signals were visualized using chemiluminescent nucleic acid detection module (Thermo Scientific) according to the manufacturer's protocol.

## Results

### Genome-wide TSS and operon maps

To obtain an overview of the cold response of the *M. psychrophilus* R15 primary transcriptome, two different RNA sequencing approaches were combined: dRNA-seq for identification of the 5′-ends and strand-specific whole-transcript sequencing. Three cDNA libraries, 5′-end (+) library, 5′-end (−) library and strand-specific whole-transcript library, were individually constructed from the same rRNA-depleted RNA pool extracted from the mid-log phase cultures grown at 18°C and 8°C.

Libraries were sequenced on an Illumina HiSeq platform. Only ≤1% of the resulting reads mapped to rRNA in the whole-transcript libraries and 2–17% of rRNA residual in the 5′-end libraries, respectively (Supplementary Table S1), indicating a high rRNA depletion efficiency, a technological challenge for archaeal RNA-seq^13^,^31^. To evaluate the accuracy of RNA-seq libraries for mRNA quantification of abundance, RPKM values of the detected 1.37% ERCC spike-in RNA reads were determined. They correlated well with the spike-in concentration (r = 0.968, Supplementary Figure S1). The trimmed lengths of mapped reads in cDNA libraries varied from 32 to 97 bp. In total, ~41 M unique mapped reads for dRNA-seq libraries and ~63 M for whole-transcript sequencing libraries were collected (Supplementary Table S1).

**Figure 1:**
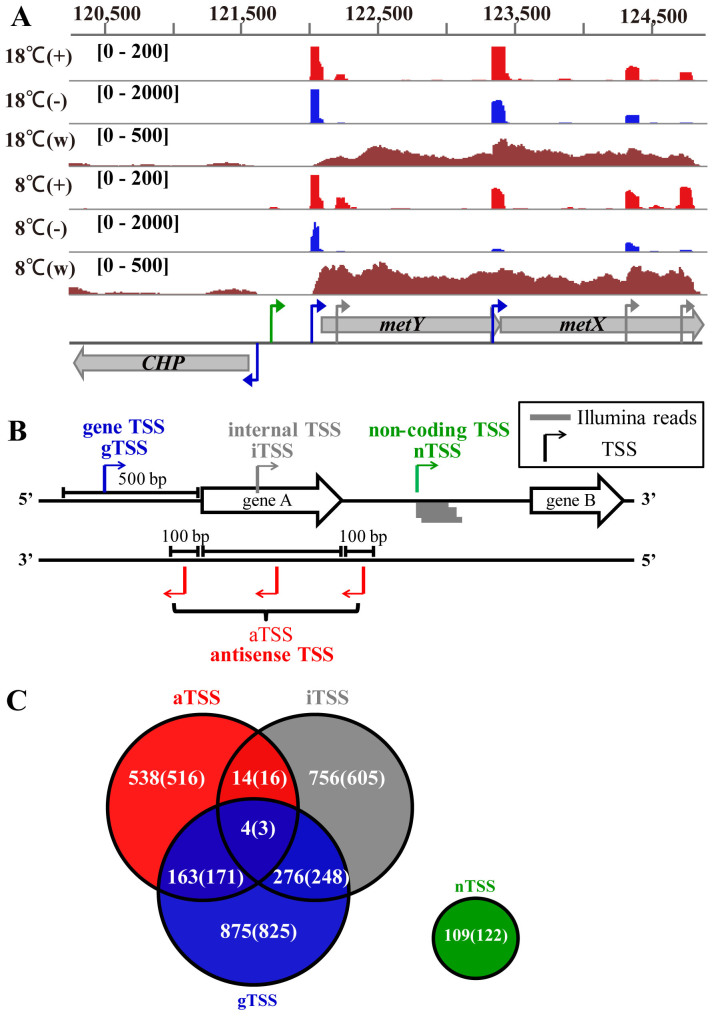
Identification of the primary transcription start sites (TSSs) in *M. psychrophilus* R15. Using an integrated approach including differential RNA-sequencing (dRNA-seq) and whole-transcript sequencing, TSSs were identified in the temperature-responsive transcriptome of R15 growing at 8 and 18°C. (A) cDNA reads mapped to the *metYX* operon from dRNA-seq (+) and (−) libraries are shown as red and blue, respectively, and those from the whole-transcript (w) sequencing libraries are shown in brown. (+) indicates a 5′-end library for primary transcripts and (−) indicates a 5′-end library for both primary and processed transcripts. Blue, green, and grey arrows indicate the gene transcription start sites (gTSS), intergenic region TSS (nTSS), and gene-internal transcription TSS (iTSS), respectively. (B) Diagram of TSS categories based on the expression and gene architecture: gene (g), internal (i), antisense (a), and non-coding (n) RNA. Grey lines represent the Illumina reads. (C) Venn diagram of the TSS numbers detected in 18 (outside the parenthesis) and 8°C (inside the parenthesis) libraries. Many TSSs affiliated to multiple categories. A total of 2506 TSSs were identified in the 8°C library, and 2735 TSSs were identified in the 18°C library.

The cDNA sequences generated from dRNA-seq libraries were enriched at the +1 site of transcription. This enrichment (≥10 reads enriched in the 5′-end (+) libraries) and other plausible criteria described in Methods helped TSS identification. By integrating cDNA mappings from the 5′-end (+) and (−) libraries and the whole-transcript libraries, the sequencing approaches identified the transcription start sites (TSSs) for many of the operons. Examples of TSS identifications are shown in Figure 1A. TSS categories (Figure 1B) were further refined through mapping the whole-transcript cDNA reads to the corresponding 5′-end reads. Manual checking reassigned about 100 gene TSSs (gTSSs) (Supplementary Table S3). Most of the mRNAs in the whole-transcript libraries lacked the 20–30 nt from their proximal 5′ terminus, as reviewed by Wang *et al*.^32^. This indicates that the canonical whole-transcript sequencing did not obtain the precise 5′ end (TSS) for most mRNAs.

We detected expression of 2925 and 3068 out of the 3167 original annotated ORFs in the 18°C- and 8°C-whole-transcript cDNA libraries, respectively (Supplementary Table S4). Combining DOOR database^24^ and Rockhopper methods^25^ and manual curation as described in Material and Methods, 671 operons (≥2 genes) that comprise 2006 ORFs were predicted (Supplementary Figure S2, Supplementary Table S5). The largest operon was the ribosomal protein complex that comprised of 23 genes with a single gTSS (Supplementary Figure S2A). However, another large operon for archaeal exosome, an RNase complex to mediate 3′–5′ degradation of RNAs^33^, contained internal TSSs (Supplementary Figure S2B), suggesting a dynamic transcription of suboperons.

By annotation of cDNAs with ≥10 reads enriched in the 5′-end (+) libraries, 2506 and 2735 non-redundant TSSs were identified in the 8°C- and 18°C- transcriptomes, respectively (Figure 1C). Those included 1680 gTSSs for 1534 annotated ORFs (Supplementary Table S3), 195 TSSs for non-coding RNAs (nTSSs) present in 125 intergenic regions (IGRs) (Supplementary Table S6), 1110 aTSSs (Supplementary Table S7) in 908 ORFs and 1440 iTSSs (Supplementary Table S8) inside 1068 ORFs. Promoters including transcription factor IIB recognition element (BRE) and TATA box (Supplementary Figure S3) could be predicted at approximately 19–22 nt from the TSS in 81.6% of the primary transcripts.

### Multi-TSSs and temperature-responsive TSS selection of a gene resulting alternative 5′ UTR mRNA isoforms

**Figure 2:**
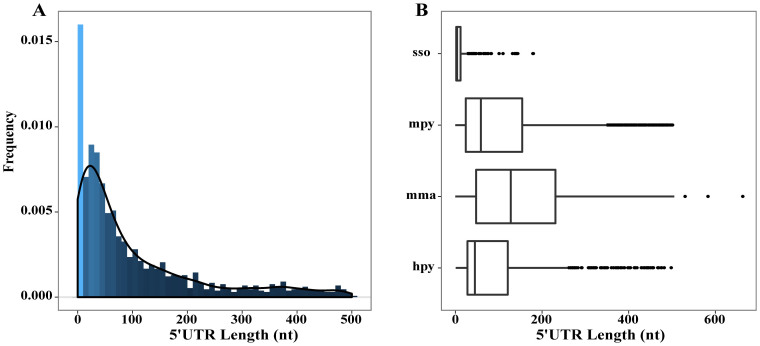
5′ UTR length distribution in the *M. psychrophilus* R15 transcriptomes. (A) Distribution of the 5′ UTR lengths in the R15 primary transcripts. Here, 15.56 and 16.01% transcripts were leaderless (UTR <10 nt) in the 8 and 18°C 5′-end libraries, respectively. (B) Box plots for genome-wide lengths of the 5′ UTRs distributions in *Sulfolobus solfataricus* (sso) P2, *Methanolobus psychrophilus* (mpy) R15, *Methanosarcina mazei* (mma) Gö1, and *Helicobacter pylori* (hpy 26695). The box indicates the range from the lower to the upper quartile, and the line inside each box refers to the median. Extreme outliers are depicted by dots, showing that the UTRs distributed at lengths of 300–500 nt are common in R15.

We detected 1318 and 1247 gTSSs in the 18°C- and 8°C-transcriptomes of R15, respectively. Some TSSs were confirmed by 5′-RACE (Supplementary Figure S4), indicating the single-nucleotide resolution of dRNA-seq identified TSSs in this work. The 5′ UTRs of transcripts were identified as the regions between the TSS and the start codon of an ORF. The 5′ UTR lengths of the R15 primary transcripts were mainly 20–150 nt (Figure 2A), with a median length of 55 nt (Figure 2B) and the longest length of 497 nt. 15.6% and 16% of transcripts were leaderless (5′ UTR <10 nt) at 8°C- and 18°C, respectively. About 51% of the 5′ UTRs were >50 nt in length, and 24% were >150 nt. Most of the ribosomal protein mRNAs had longer 5′ UTRs, with an average of 165 nt (Supplementary Table S3).

**Figure 3:**
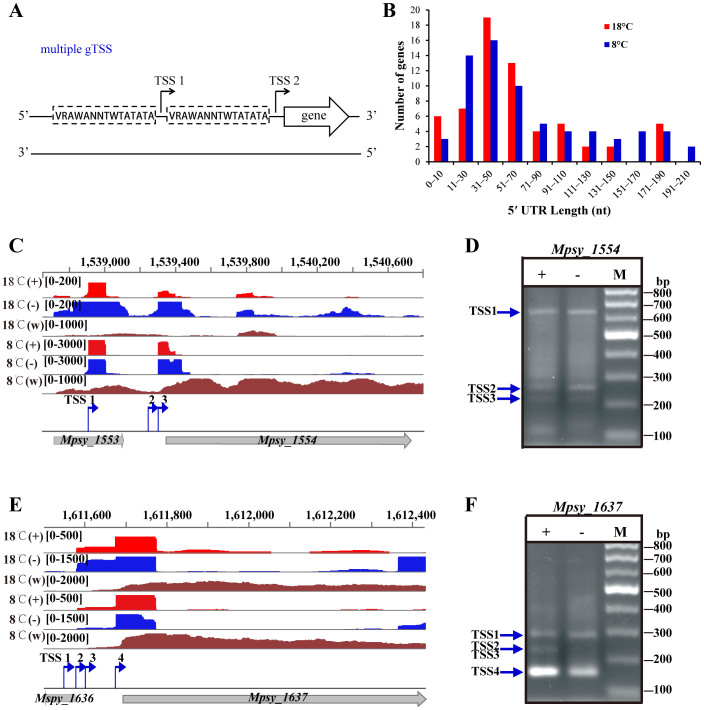
Multiple promoters and TSSs identified for representative ORFs in *M. psychrophilus* R15. Multiple TSSs were common for R15 genes and appeared to be related to changes in the 5′ UTR lengths at different temperatures. (A) Schematic of the promoters and gTSSs used by a given ORF. The broken line frames identify potential promoter features; arrows, TSSs. (B) A bar diagram showing the distributions of 5′ UTR lengths in the 18°C (Red bars) and 8°C (blue bars) transcriptomes. (C) and (E) IGV genome browser mapped the reads of the dRNA-seq (+) and (−) libraries at 8 and 18°C and revealed two TSSs for the cell surface protein gene (Mpsy_1554) and four TSSs in a XRE family transcriptional regulator (Mpsy_1637). (w), whole-transcript RNA-seq read mapping. Read scales are shown in brackets. (D) and (F) 5′-RACE assays validated the dRNA-seq detected TSSs for Mpsy_1554 and Mpsy_1637. Agarose gels (2.5%) showed the RT-PCR products that were treated with (+) or without (−) tobacco acid pyrophosphatase (TAP) indicted above the lanes. M shows DNA ladders. Blue arrows point the TSSs detected by 5′-RACE. Oligos used in 5′-RACE are listed in Supplementary Table S2.

**Figure 4:**
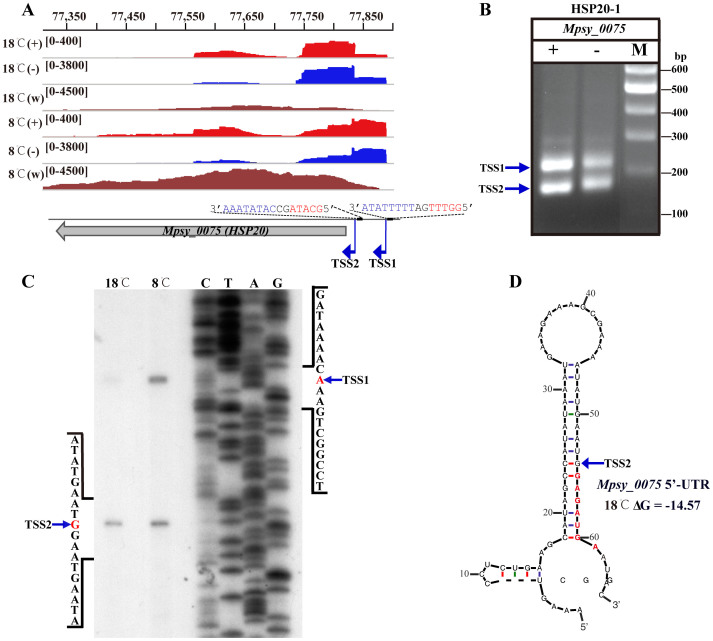
Cold-induced gTSS selection by an Hsp20 gene resulting in alternative 5′ UTR mRNA isoforms. (A) IGV genome browser showed that reads of the dRNA-seq (+) and (−) libraries of a heat shock protein Hsp20 gene (Mpsy_0075) were mapped more to the upstream TSS1 in the 8°C-5′ end library and more to the downstream TSS2 in the 18°C-5′ end library. Red and blue bases at the upstream of TSS1 and TSS2 represent likely BRE elements and TATA boxes, respectively. cDNAs from the whole-transcript libraries (w) covered the ORF were remarkably increased in the 8°C-library. Reads of the dRNA-seq (+) and (−) libraries are shown in red and blue curves, respectively. Those of the whole transcript are in brown. Read scales are in brackets. (B) 5′-RACE confirmed the two TSSs of Mpsy_0075 identified by dRNA-seq. (C) Primer extension assay of Mpsy_0075 mRNA in the 8 and 18°C 5′-end libraries. 10 μg mRNA from each library was used, the oligonucleotide was complementary to <300 nt downstream the start codon. dRNA-seq identified TSS1 and TSS2 in the two 5′-end libraries were specified. (D) Mfold predicted the secondary structures of the 65 nt-5′ UTR identified in the 8°C library. Arrows indicates the TSS2 used at 18°C. Bases in red represent the predicted RBS.

Interestingly, multiple gTSSs were identified for 219 ORFs (Supplementary Table S9, Figure 3A). Strikingly, 84 ORFs exhibited temperature-dependent gTSS, indicating a temperature-induced gTSS-selection (Supplementary Table S9). For these ORFs, transcripts with different length 5′ UTR, i.e. RNA isoforms were observed. The leaderless mRNAs (with <10 nt 5′ UTR) were more abundant in the 18°C-transcriptome, while longer 5′ UTR from 120 to 250 nt were synthesized more frequently at 8°C (Figure 3B). Moreover, for some of these 84 genes, there was a remarkable increase in the lengths of their 5′ UTRs at 8°C (Supplementary Figure S5). For example, Figures 3C and 3E show dRNA-seq detected multi-gTSSs which were verified by 5′-RACE (Figures 3D and 3F). An Hsp20 gene (Mpsy_0075) exhibited a notable temperature-responsive gTSS-selection, by preferentially using the upstream promoter and TSS at 8°C (Figure 4A and 4B) which was confirmed by primer extension assay (Figure 4C). This resulted in more mRNA isoform with a 65-nt 5′ UTR at 8°C and more with a 12-nt 5′ UTR at 18°C. A secondary structure was predicted in the 65-nt 5′ UTR (Figure 4D), in which ribosome binding site (RBS) was buried and may hinder translation. Another Hsp20 encoding gene (Mpsy_0869) also exhibited the temperature-related gTSS-selection (Supplementary Figure S6).

### Cold-induced sRNAs in the intergenic regions (IGRs)

**Figure 5:**
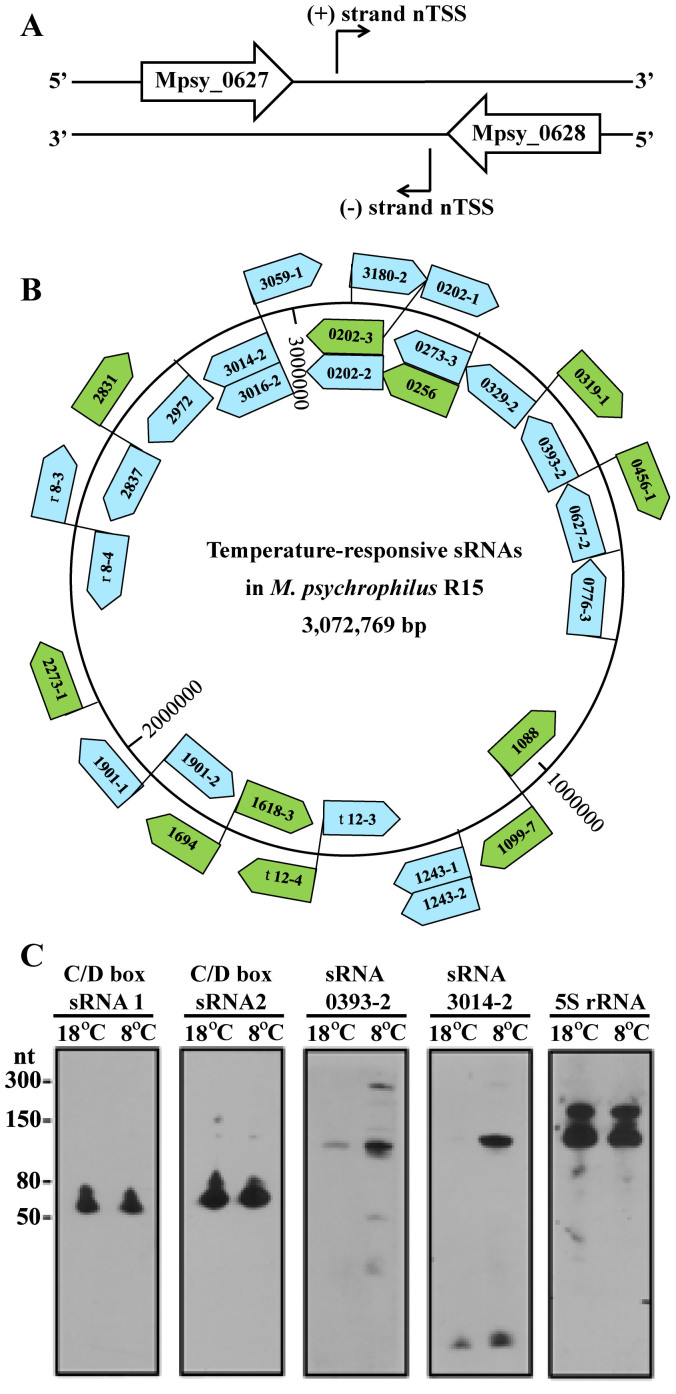
Non-coding small RNAs in the 5′-end transcriptomes of R15. (A) Schematic of the multiple nTSSs located in one intergenic region (IGR) as the mutual antisense transcripts. (B) Genome locations of the representative differentially expressed sRNAs in response to temperature changes. Inside the bullets are the denoted serial numbers of the small RNAs that are defined as the upstream locus number-sRNA number. Bullets indicate the transcription direction of each sRNA. Green bullets indicate RNA that showed ≥4-fold less expression at 8°C than at 18°C. Blue bullets indicate RNAs that showed ≥35-fold more expression at 8°C than at 18°C. t, tRNA; r, rRNA. (C) Northern blot analysis verified the transcriptions of C/D box sRNAs and cold-induced sRNAs. sRNAs and the growing temperatures of their expressions are indicated at the tops of the gels. 5S rRNA is included as the control.

A total of 195 nTSSs were detected in 129 IGRs (Supplementary Table S6). They represented noncoding small RNAs that could exert post-transcriptional regulation of gene expression. 43 IGRs contained ≥2 sRNAs, and some were mutual antisense sRNAs (Figure 5A). sRNAs, including some tRNAs, were among the ten most abundant transcripts in both libraries (Supplementary Table S10), especially the signal recognition particle RNA (SRP RNA, also known as 7S RNA). The abundance of some nTSSs increased dramatically (up to >1000-fold) in the 8°C library (Supplementary Table S6, Figure 5B). The cold-induced expression of some sRNAs was confirmed by Northern blot analysis (Figure 5C). Remarkably, nine highly conserved sRNAs were all induced by cold, and possessed a conserved motif, tentatively named the ‘cold box’, upstream of their promoters (Supplementary Figure S7). These results suggest that sRNAs are involved in the cold adaptation of the archaeon.

dRNA-seq also identified 49 gTSSs for 40 tRNA genes, and many functional sRNAs including a SRP RNA, an RNase P, three C/D box small RNAs, and two group II introns (Supplementary Table S11). Interestingly, dRNA-seq detected the expression of tRNA-Pyl gene (Supplementary Figure S8A), a dedicated pyrrolysine tRNA (tRNA-Pyl) responsible for inserting the necessary pyrrolysine residue during translation of the methylamine:corrinoid methyltransferases^34^. The previous automatic annotation failed to identify the tRNA-Pyl in the R15 genome^6^, most probably because of its distant genomic location from the aminoacyl-tRNA synthetase gene (Supplementary Figure S8B). Based on this finding, six methylamine:corrinoid methyltransferase encoding genes were re-annotated (Supplementary Figure S8C, Table S12). These genes had been disrupted by the pyrrolysine-encoding amber stop codon in automatic annotation^6^.

### Antisense transcriptions

1110 antisense transcription start sites (aTSS) that complemented 908 protein encoding genes (28.7% of total ORFs) of R15 were detected in the libraries, and promoters were predicted for 622 aTSSs (Supplementary Table S7). Supplementary Figure S9A showed the reads mapping diagrams of aTSS expressions.

As shown in Supplementary Table S7, the majority of the aTSSs overlapped internal regions of the sense transcripts, and 245 (22%) and 111 (10%) aTSSs overlapped 5′ and 3′ UTRs of the complementary mRNAs, respectively.

For many genes, the temperature-responsive abundances of aTSSs and their targeted mRNAs were either positively or negatively correlated (Supplementary Figure S9B). Expression of aTSS for genes encoding the enzymes key to methylotrophic methanogenesis, *mtaB* (Mpsy_0909), *mttC* (Mpsy_1686), *cdhE* (Mpsy_2895), *hdrBA* (Mpsy_2939, 2940), and *mtaC* (Mpsy_3032) were upregulated at 8°C, while the transcripts of these genes were reduced at 8°C (Supplementary Table S4). This suggests that antisense-based post-transcriptional regulation may play a role in the response of methanogenesis to cold.

### Gene-internal TSS

A total of 1068 genes possessed internal TSS, and 753 iTSSs were detected in the 18°C library and 601 in 8°C library. Most of iTSSs were detected at relatively lower abundance than the gTSS (Supplementary Table S8).

About 25% of iTSSs were found located near the 5′ end of the transcript (Supplementary Figure S10A) and some helped identify the precise start codon of a gene and the exact boundary of an ORF. For instance, iTSS identification refined the automatic annotation-generated atypical 5′-extension in Mpsy_1484 (DNA polymerase PolB) and Mpsy_2175 (F_420_-ligase) and re-defined the exact boundaries of the ORFs (Supplementary Figure S10B). In this work, a total of 188 ORFs were re-annotated, including four newly found ORFs (Supplementary Table S12). 51 ORFs were corrected through iTSS identification near their 5′ ends.

Approximately 30% of the identified iTSSs were located at the last quarter of the transcripts, and majority of them were also detected as the gTSSs of their downstream ORFs, indicating a concerted transcription between the neighboured genes in the condensed genomes. For example, two iTSSs located at the 3′ end of Mpsy_1892 might be the gTSSs of the downstream Mpsy_1891, implying coordinate expression of the operons for Mpsy_1891 and Mpsy_1892 (Supplementary Figure S10C).

Interestingly, iTSSs were detected for most signal transduction histidine kinase genes, suggesting an environmental clue-induced gene internal transcription. For example, an iTSS2 at position 26,651 in Mpsy_0031 would produce a truncated protein retaining only the histidine kinase (HisKA) and the receiver (REC) domains but deleting the PAS/PAC sensor-domains (Supplementary Figure S11A). These domains might have a regulatory function in the phosphorylation of the two component system composed by the protein^35^.

## Discussion

An atlas of the genome-wide transcription start sites would provide insights into the primary transcriptome for an organism at a given spatio-temporal condition^13^,^14^,^15^,^16^,^17^. This study has identified TSSs for 48.4% of the total annotated ORFs or 91% predicted operons of the psychrophilic methanoarchaeon R15. Multiple TSS per gene and cold-responsive TSS indicate a dynamic transcriptome in this archaeon, thus providing a new avenue for exploring archaeal cold adaption and the coordination between transcriptional and post-transcriptional regulation.

Precise TSS identification reveals that, similar with another methanoarchaeon *M. mazei*^13^, the majority of the mRNAs in *M. psychrophilus* R15 possess long 5′ UTRs of 20–150 nt. By providing targets of non-coding regulatory RNAs through cis- or trans-actions, large 5′ UTRs provide potential regulatory elements for post-transcriptional regulation. Specifically, 5′ UTRs are predicted to respond to ambient temperatures because of temperature-sensitive base-pair formation^36^. An example is the *E. coli* mRNA of cold shock protein CspA, which has a 160 nt-5′ UTR that behaves like a RNA thermometer^9^. In addition, large 5′ UTRs may act as riboswitches in gene regulation^37^. Eukaryotic mRNAs generally have long 5′ UTRs, with the average length of ~100 to ~200 nt^11^, and they are of the biological importance in controlling transcript stability, intracellular localization, differential translation and microRNA mediated gene regulation^38^. Oncogenes, tumor suppressors and others associated with cell proliferation all tend to express atypically long and complex 5′ UTRs that are involved in subtle regulations of the corresponding genes^39^.

This study found that 14% of R15 genes used multiple TSSs with individual promoters (Figure 3, Supplementary Table S9). In prokaryotes, multiple TSSs have been reported in only a few bacteria, such as *E. coli*^40^ and the cyanobacterium *Anabaena*^17^. In contrast, 10–18% of mammalian genes are reported to use multiple TSSs, especially oncogenes, tumor suppressors and genes associated with cell proliferation^12^. Transcription of a gene from multiple TSSs would produce mRNA isoforms with alternative 5′ UTRs, which contribute to the development of carcinogenesis. Alternative 5′ UTRs for *E. coli* *infA* transcript also shows regulatory functions in translation efficiencies and mRNA stability^41^. Remarkably, 84 genes in R15 possessed cold-responsive selection of gTSSs and promoters and would be expected to transcribe different mRNA isoforms with alternative 5′ UTRs at different temperatures, like the Hsp20 genes. Though a number of transcriptomic studies on prokaryotes have indicated that many genes are differentially expressed in the cold, a conserved motif or cold box has not been found proximal to the prompters^2^,^3^,^4^,^10^. Therefore, it is believed that post-transcriptional regulation, including mRNA stability and translation efficiency, make major contributions to cold adaptation of living organisms^7^. However, the prevalence of alternative 5′ UTRs generated by cold-responsive gTSS selection (transcriptional regulation) identified in this work suggests that coordinated regulation mechanism between transcription and post-transcription may be of importance in the cold adaption of methanoarchaea.

Non-coding small RNAs (sRNA) have been identified in all the three domains of life and represent important players in post-transcriptional regulation. However, archaeal sRNAs are poorly characterized^42^. 195 sRNAs were identified in this work (Supplementary Table S6), some are categorized in the most abundant transcripts in R15 (Supplementary Table S10). 40% of the sRNAs were differentially expressed in the cold, and nine conserved sRNAs with a predicted cold box upstream the promoters were dramatically induced at 8°C. These findings indicate that sRNAs play important roles in cold-responsive regulation in this archaeon.

Identified functional sRNAs include signal recognition particle (SRP) RNA, which is an essential component of SRP. In Archaea, it delivers proteins to the plasma membrane^43^. The SRP RNA in R15 is among the most abundant primary transcripts at both temperatures (Supplementary Table S10). This can be related to the 83 proteins that contain signal peptides in *M. psychrophilus* R15 including S-layer proteins. In addition, C/D box sRNAs involved in 2-*O*-methylation of rRNA and tRNAs are also abundantly expressed, and their associated proteins, such as fibrillarin (Mpsy_1937), Nop5 (Mpsy_1936) and L7Ae (Mpsy_2863), are all expressed. Nucleotide modification of rRNAs may play a role in temperature adaptation, at least in thermophiles. For instance, ribose methylation levels increase in *S. solfataricus* RNAs at high temperatures^44^. Because there are fewer C/D box sRNAs in *M. psychrophilus*, post-transcriptional modifications of rRNAs may be less important to psychrophiles.

Antisense transcription, which has been found in the majority of prokaryotic and eukaryotic genomes^45^, can affect the overlapping sense transcripts via a double-stranded RNA-dependent mechanism. An RNAi-like function displays mutual exclusion between the sense and paired antisense transcripts. asRNA-driven mRNA degradation plays a role in post-transcriptional regulation in methanogenic Archaea^46^. Antisense transcription was found in 28.7% of total R15 ORFs, and many were upregulated at 8°C (Supplementary Table S7), indicating their involvements in the cold-adaptation of R15.

In conclusion, this study reveals an unexpectedly dynamic transcriptome in a psychrophilic archaeon in response to cold, shedding light on transcriptional and post-transcriptional regulation in archaeal cold adaption. The findings of this study are also of evolutionary significance. As the *cspA* of *E. coli* employs the long 5′ UTR-transcript to achieve the post-transcriptional regulation and alternative 5′ UTRs are a major regulatory element in mammalian gene expressions, the large and alternative 5′ UTRs found in this psychrophilic methanogenic archaeon might hint at an evolutionary conservation between methanogenic Archaea and eukaryotes and demonstrate the importance of post-transcriptional regulation mediated by the 5′ UTRs in archaeal gene expression.

## Supplementary Material

Supplementary Information Global mapping transcriptional start sites revealed both transcriptional and post-transcriptional regulation of cold adaptation in the methanogenic archaeon Methanolobus psychrophilus
