# Supplementary material for: Global mapping transcriptional start sites revealed both transcriptional and post-transcriptional regulation of cold adaptation in the methanogenic archaeon Methanolobus psychrophilus
Source: Sci Rep. 2015 Mar 18;5:9209. doi: 10.1038/srep09209 (PMC5378194; doi:10.1038/srep09209)
Supplement: Supplementary Information — Global mapping transcriptional start sites revealed both transcriptional and post-transcriptional regulation of cold adaptation in the methanogenic archaeon Methanolobus psychrophilus [file srep09209-s1.pdf]

Global mapping transcriptional start sites revealed both transcriptional and post-transcriptional regulation of cold adaptation in the methanogenic archaeon *Methanolobus psychrophilus*

Jie Li[1,#], Lei Qi[1,#], Yang Guo[2], Lei Yue[1], Yanping Li[2], Weizhen Ge[2], Jun Wu[2,*], Wenyuan Shi[3] and Xiuzhu Dong[1,*]

[1] State Key Laboratory of Microbial Resources, Institute of Microbiology, Chinese Academy of Sciences, No.1 Beichen West Road, Beijing 100101, People's Republic of China

[2] Novogene Bioinformatics Institute, 21st Floor, Jinma building B area, Xueqing Road, Beijing 100083, People's Republic of China

[3] Department of Microbiology, Immunology and Molecular Genetics, University of California, 10833 Le Conte Avenue, Los Angeles, CA90095, USA.

* To whom correspondences should be addressed. Xiuzhu Dong, Tel: +86 10 6480 7413; Fax: +86 10 6480 7429; Email: dongxz@im.ac.cn. Correspondence may also be addressed to Jun Wu, Tel: +86 10 8283 7567; Fax: +86 10 8283 7567; Email: wujun@novogene.cn.

# the authors contributed equally to this work.

Supplementary figures and table legends:

Figure S1. Correlation between RNA-seq determined RPKM of the spike-in RNAs and the final concentration in RNA pools of 18°C culture (A) and 8°C culture (B). External RNA Controls Consortium (ERCC) spike-in control mixes (Ambion) was used as internal control. Concentrations of the added ERCC RNA and the RPKMs determined by whole-transcript sequencing were shown at x- and y-axis, respectively. The correlation coefficients, indicating the accuracy of the RNA-seq detection for RNA levels. r, correlation coefficient calculated by “pearson” method; R^2, the square of r; tau, correlation coefficient calculated by “kendall” method; n, numbers of the ERCC RNA with RPKM >2 in RNA-seq detection.

Figure S2. Examples of operon determination through integration of primary gTSSs and whole-transcript sequencing. Based on the prediction results of both DOOR database and Rockhopper method, operons were defined as the DNA regions with a continuous coverage by the whole-transcript sequencing reads and a mapped upstream gTSS. The schematic diagrams of the operons were generated by IGV genome brower. (**A**) Representative cDNA mappings to the ribosomal protein operon consisted of Mpsy_1124 (*rpl15*) to 1146 (*rpl3*) that are encoded by lagging strand. Only one gTSS was detected upstream of Mpsy_1146 in the 5′-end (+) libraries at two temperatures, and continuous mapping in the whole transcript sequencing library (w) was found on the operon. (**B**) Representative cDNA mappings to the exosomal operon retrieved from 5′-end (+)/(−) and whole-transcript (w) transcriptomes of 18 and 8°C libraries showed temperatue-induced suboperon organization. Primary gTSSs (blue

arrows) were detected for Mpsy_2722 (*rpl15e*), Mpsy_2725 (*rpp14*), Mpsy_2726 (*psmA*), Mpsy_2732 (*rpoP*), Mpsy_2735 (*pfdB*), Mpsy_2737 (*paak*), Mpsy_2738 (ACT domain) and Mpsy_2739 (DUF2103) in both 18 and 8°C 5′-end (+) library, and coexpression of Mpsy_2722 (*rpl15e*) to 2725 (*rpp14*), Mpsy_2726 (*psmA*) to 2732 (*rpoP*) were detected in 18°C whole-transcript library. Whereas, the gTSS of Mpsy_2726 (*psmA*) was increased in 8°C 5′-end (+) library, and coexpression of Mpsy_2726 (*psmA*) to 2732 (*rpoP*) was also detected in the 8°C whole-transcript library. Blue arrows, gTSSs detected in (+) library. (+), 5′-end library for primary transcripts ; (−), 5′-end library for both primary and processed transcripts. On the top of each diagram, the genome location is indicated.

Figure S3. Sequence-logo of promoter regions based on dRNA-seq identified TSSs. The upstream regions of the TSSs (up to 500 nt) were aligned using MEME and consensus visualized with Web Logo. Upstream sequences were aligned for (**A**) 1371 out of 1680 gTSSs (81.6%), (**B**) 622 out of 1110 aTSSs (56%), (**C**) 305 out of 1440 iTSSs (21.2%) and (**D**) 22 out of 190 nTSSs (11.6%), respectively. Promoter motifs (TATA boxes located at −23 to −30 nt and BRE boxes centered at −35 nt) are predicted. MEME searched promoter sequences for gTSS, nTSS, aTSS and iTSS are listed in the Promoter column in Dataset S1, Dataset S4, Dataset S5 and Dataset S6, respectively.

Figure S4. 5′-RACE assay verified selected gTSSs detected by dRNA-seq. Agarose gels (2.5%) showed RT-PCR products that were treated with (+) or without (−) tobacco acid pyrophosphatase (TAP). Gene annotation and locus number are

indicated in each gel. The primers used in 5′-RACE are listed in Supplementary Table S2. Arrows indicate the 5′-RACE bands that confirmed the dRNA-seq identified TSSs. M, DNA molecular markers and the lengths are shown on the right.

Figure S5. Changing ratios of 5′ UTR length in each of the 84 genes of R15 showing temperature-related gTSS selection. By dividing the length (nt) difference of 5′ UTR in 18°C 5′- vs. 8°C 5′-end libraries over the relative shorter length at each temperature for each gene, the changing ratios of the 5′ UTR lengths are calculated for the 84 genes that exibited temperature-related gTSS selection in 18°C 5′- vs. 8°C 5′-end libraries. Blue bars repesent the genes with increased 5′ UTR length at 8°C, and red bars repesent the genes with increased 5′ UTR length at 18°C.

Figure S6. Multiple gTSSs identified in a Hsp 20 gene. (**A**) IGV genome browser showed the dRNA-seq reads mapping detected in 5′ (+)/(−) libraries of a heat shock protein Hsp20 gene (Mpsy_0869). That revealed three TSSs both in 18 and 8°C 5′-end libraries, and reads mapped more to upstreamed TSS2 and TSS3 in 18°C library. cDNAs from the whole-transcript sequecing library (w) covered the ORF were remarkaly increased in the 8°C library. (**B**) 5′-RACE assay verified dRNA-seq detected three gTSSs. Agarose gels (2.5%) showed the RT-PCR products that were treated with (+) or without (−) tobacco acid pyrophosphatase (TAP) indicted above the lanes. Blue arrows point the TSSs. Oligos used in 5′-RACE are listed in Supplementary Table S2. (**C**) Primer extension assay of Mpsy_0869 mRNA in 8 and

18°C cultures. 10 µg mRNA from each cultures was used, and the oligos complemetary to <50 nt downstream the start codon was used and listed in Supplementary Table S2. dRNA-seq identified three TSSs in the two 5′-end libraries were specified in the two sides of the gel. (**D**) Mfold predicted the secondary structures of the Hsp20 5′ UTR generated from TSS1. Arrow indicates the TSSs, and bases in red represent the predicted RBS.

Figure S7. A group of conserved cold-induced sRNA candidates in R15. (A) Alignment of nine conserved sRNAs. Conserved promoter elements (BRE and TATA box) and dRNA-seq determined TSSs are indicated. Inside the red broken line frame is a conserved motif upstream the promoter elements, tentatively named “cold box”. (**B**) Northern blot analysis of the temperature-responsive differential expressions of the conserved sRNAs. Using four probes that each target one of the nine sRNAs detected all the nine. Probed sRNAs are indicated on the top of the lanes.

Figure S8. dRNA-seq identified a tRNA-Pyl in the R15 transcriptome which facilitates amending automatic annotations on methylamine methyltransferase genes. (**A**) cDNA mapping to tRNA-Pyl gene showed the gTSS upstream the mature tRNA-Pyl (72nt, grey bullet). On the top shows the genome location of tRNA-Pyl gene. (**B**) Genome architecture of the genes for tRNA-Pyl synthesis. (**C**) Schematic diagram of the genome architecture by automatic annotation showing that genes

encoding methylamine:corrinoid methyltransferases are disrupted by the pyrrolysine encoding amber stop codon. Solid line framed are the corrected genes by joining the automatic annotated gene fragments by considering UAG stop codon to be translated to pyrrolysine. This is indicated by the downstream characteristic PYLIS (pyrrolysine insertion sequence) sequence. Those inside the broken line frames are the genes should be joined according to multi-alignments. Open bullets indicate putative genes. Detailed gene re-annotations were listed in Table S2.

Figure S9. Antisense transcriptions and their correlation with the complemented mRNA abundance in response to temperature change. (**A**) IGV genome browser visualized the dRNA-seq reads mapping in 18°C 5′-end (+)/(−) library, that revealed an antisense transcription (aTSS) covered a manganese transporter gene (Mpsy_0424) (upper panel) and two aTSSs covered an amino acid transporter (Mpsy_1700) (lower panel). Underneath showed the whole-transcript cDNA reads mapping (w) to the two genes. Grey bullets represent the genes and their genome locations as indicated on the diagram top. Inside the frame indicates the predicted promoter sequences, of which the blue and red bases represent TATA box and BRE. +1 specifies TSS. (**B**) mRNAs with ≥2-fold difference at abundance in 8 vs. 18°C library were retrieved from Dataset S2 for correlation analysis. Y-axis and x-axis indicate the fold changes of antisense transcription and the complemented transcript abundance in 8 vs. 18°C library, respectively. Each dot represents a pair of antisense and sense transcript, and dots distributed in phase I and III indicate positive correlations, while in phase II and

IV indicate negative correlations.

Figure S10. Internal transcription and that generated putative possible functions. (**A**) Location of the iTSSs inside an ORF. By equally dividing an ORF into twenty portions (x axis), numbers of the iTSSs location (y axis) in each portion is counted. 25% iTSSs situate at the proximal N-terminal 1/4 region and some are the authentic gTSSs of an ORF, so facilitates re-annotations for 51 ORFs (Dataset S9). 30% iTSSs situate at the 1/4 region proximal to C-terminal, those can be a gTSS of the downstream ORF, or to generate a suboperon. (**B**) Representative iTSSs at N-terminus were the authentic gTSSs of the ORF that enabled correction of the automatic annotation for DNA polymerase Pol 2 (Mpsy_1484, upper panel) and a F_{420}-ligase (Mpsy_2175, lower panel). Grey bullet, automatic annotated ORF, blue bullet, re-annotated ORF. (**C**) Representative iTSSs at C-terminus acted as the gTSS of the downstream ORFs. Upper panel, two iTSSs in Mpsy_1892 were the gTSSs of Mpsy_1891. Lower panel, an iTSS in Mpsy_2875 acted as the gTSS of Mpsy_2874. Grey arrow, iTSS; blue arrow, gTSS; red arrow, aTSS; green arrow, nTSS. Inside the frame indicate the predicted promoter sequences, of which the blue and red bases represent TATA box and BRE, while the three bold red bases encode the start codon. +1 specifies TSS.

Figure S11. Internal transcription generated truncated proteins that can perform alterative functions in the multi-domain proteins. IGV genome browser visualized the dRNA-seq reads mapping in 18°C 5′-end library that revealed the internal

transcriptions (iTSSs) of two genes encoding signal transduction histidine kinase of (**A**), Mpsy_0031 and (**B**) Mpsy_0087. Underneath showed the whole-transcript cDNA (w) read mapping to the two genes. Grey bullets represent the genes and the genome locations indicated on the top of the diagram. Inside the frame indicate the predicted promoter sequences, of which the blue and red bases represent TATA box and BRE, while the three bold red bases encode the start codon. +1 specifies TSS. Predicted protein domains generated by internal transcription from the two genes were shown underneath.

Table S1. Overview of the sequencing data information

Table S2. Primers and probes used in this study

Table S3. gTSSs identified in the 18 and 8°C 5'-end libraries of *M. psychrophilus* R15 by dRNA-seq and amended with whole-transcript sequencing (Excel file)

Table S4. Differential expression of the whole-transcript of *M. psychrophilus* R15 grown at 18 vs. 8°C (Excel file)

Table S5. Operon map of *M. psychrophilus* R15 (Excel file)

Table S6. nTSSs identified in *M. psychrophilus* R15 (Excel file)

Table S7. aTSSs identified in *M. psychrophilus* R15 (Excel file)

Table S8. iTSSs identified in *M. psychrophilus* R15 (Excel file)

Table S9. Multiple gTSSs and temperature-induced gTSS selection in *M. psychrophilus* R15 (Excel file)

Table S10. Top 10 most abundant transcripts in TSS semi-quantitative analysis of R15 cultured at two temperatures

Table S11. Functional RNAs identified in *M. psychrophilus* R15 by dRNA-seq (Excel file)

Table S12. Re-annotated genes for *M. psychrophilus* R15 based on dRNA-seq identification of TSSs (Excel file)

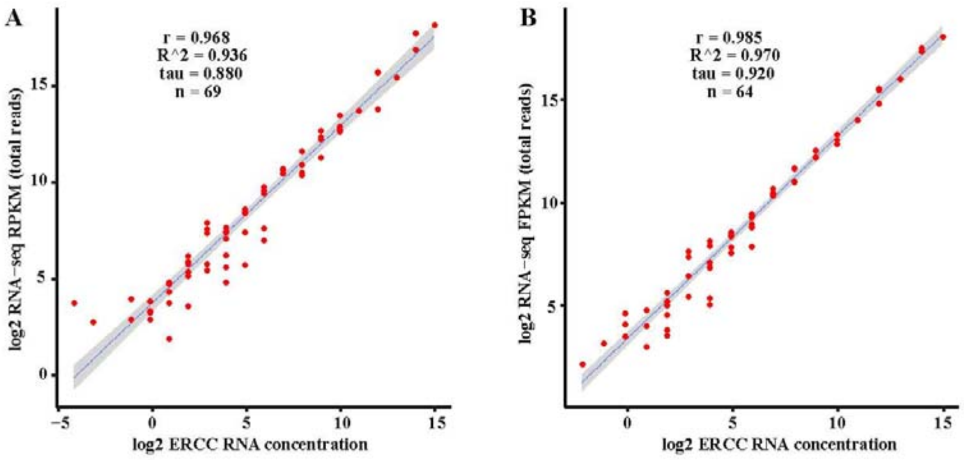
A
r = 0.968
R^2 = 0.936
tau = 0.880
n = 69
log2 RNA−seq RPKM (total reads)
log2 ERCC RNA concentration
B
r = 0.985
R^2 = 0.970
tau = 0.920
n = 64
log2 RNA−seq FPKM (total reads)
log2 ERCC RNA concentration

Figure S1

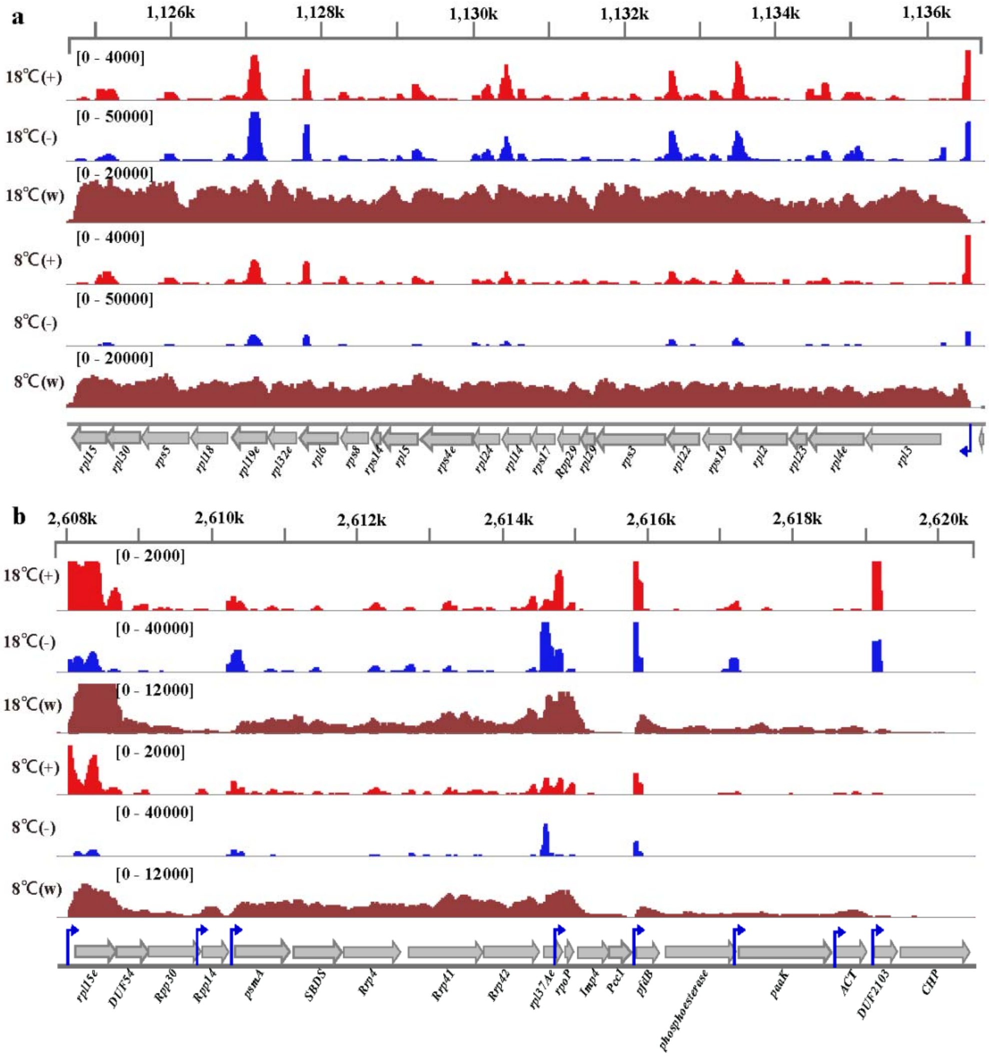
a
1,126k
1,128k
1,130k
1,132k
1,134k
1,136k
18℃(+)
[0 - 4000]
18℃(-)
[0 - 50000]
18℃(w)
[0 - 20000]
8℃(+)
[0 - 4000]
8℃(-)
[0 - 50000]
8℃(w)
[0 - 20000]
rpl15
rpl30
rps5
rpl18
rpl19e
rpl32e
rpl6
rps8
rps14
rpl5
rps4e
rpl24
rpl14
rps17
Rpp29
rpl29
rps3
rpl22
rps19
rpl2
rpl23
rpl4e
rpl3
b
2,608k
2,610k
2,612k
2,614k
2,616k
2,618k
2,620k
18℃(+)
[0 - 2000]
18℃(-)
[0 - 40000]
18℃(w)
[0 - 12000]
8℃(+)
[0 - 2000]
8℃(-)
[0 - 40000]
8℃(w)
[0 - 12000]
rpl15e
DUF54
Rpp30
Rpp14
psmA
SBDS
Rrp4
Rrp41
Rrp42
rpl37Ae
rpoP
Imp4
Pcc1
pfdB
phosphoesterase
paaK
ACT
DUF2105
CHP

Figure S2

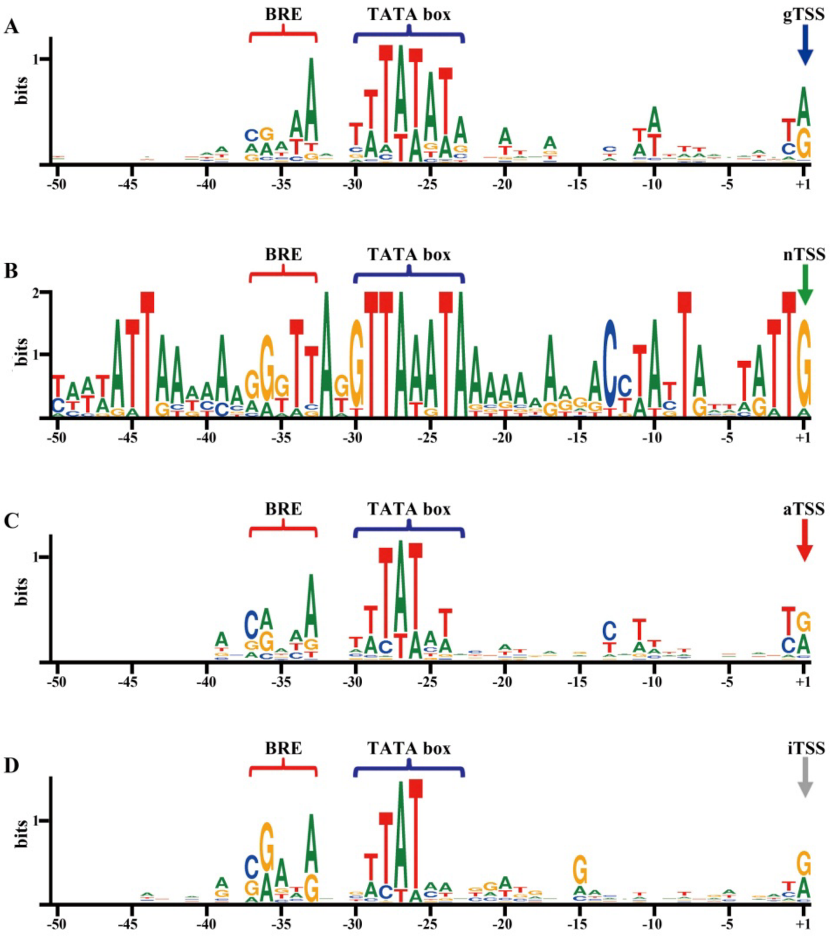
A
B
C
D
BRE
TATA box
gTSS
nTSS
aTSS
iTSS
bits
-50
-45
-40
-35
-30
-25
-20
-15
-10
-5
+1

Figure S3

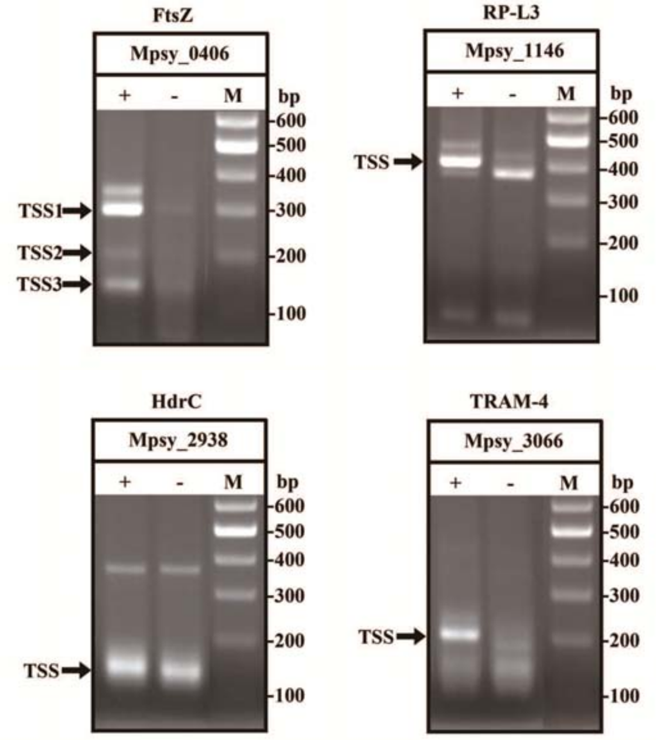
FtsZ
Mpsy_0406
+
-
M
bp
600
500
400
300
200
100
TSS1
TSS2
TSS3
RP-L3
Mpsy_1146
TSS
HdrC
Mpsy_2938
TRAM-4
Mpsy_3066

Figure S4

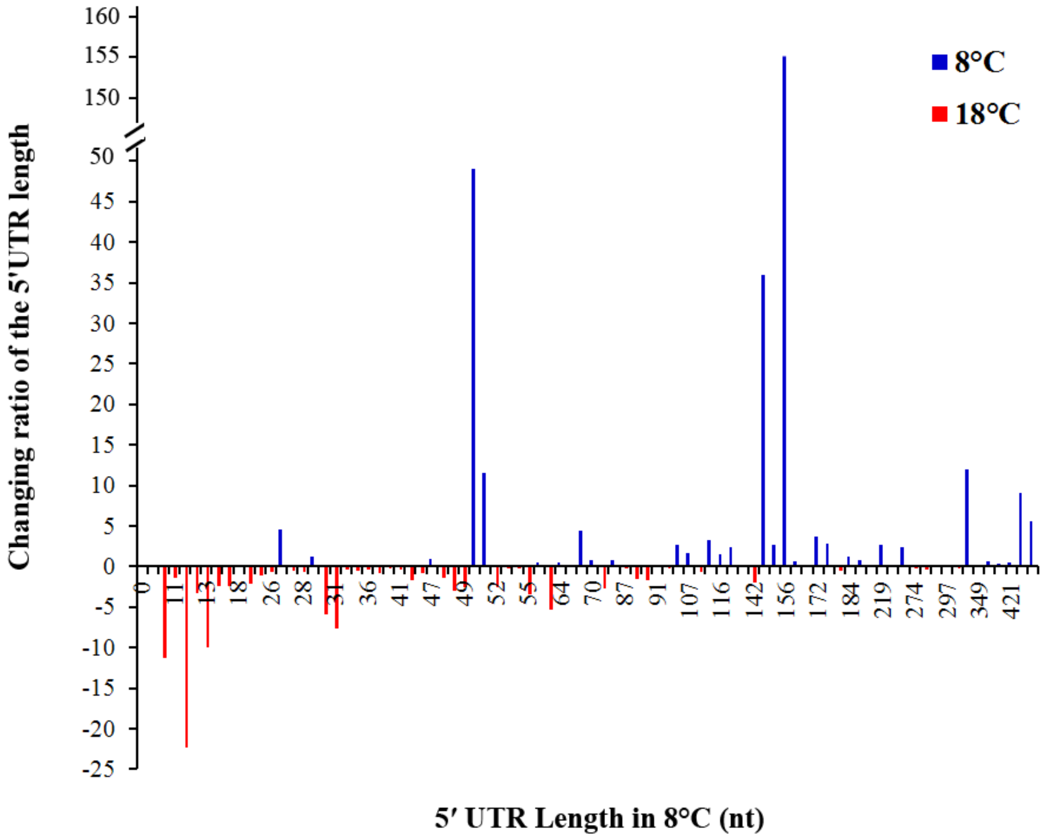

8°C
18°C
Changing ratio of the 5'UTR length
5′ UTR Length in 8°C (nt)
160
155
150
50
45
40
35
30
25
20
15
10
5
0
-5
-10
-15
-20
-25
0
11
13
18
26
28
31
36
41
47
49
52
55
64
70
87
91
107
116
142
156
172
184
219
274
297
349
421

Figure S5

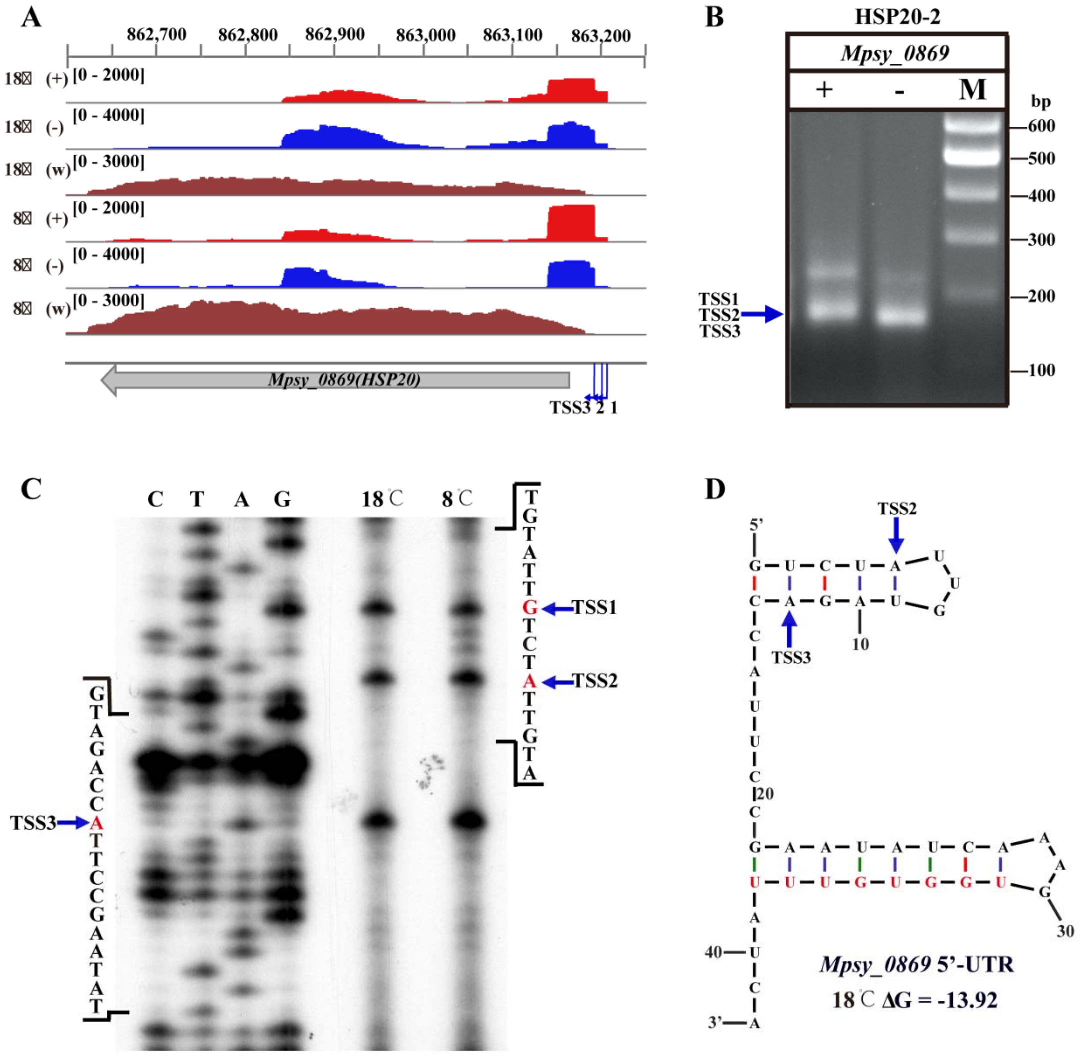
A
862,700
862,800
862,900
863,000
863,100
863,200
[0 - 2000]
[0 - 4000]
[0 - 3000]
Mpsy_0869(HSP20)
TSS3 2 1
B
HSP20-2
Mpsy_0869
+
-
M
bp
600
500
400
300
200
100
TSS1
TSS2
TSS3
C
C T A G
18℃
8℃
TSS1
TSS2
TSS3
D
TSS2
TSS3
Mpsy_0869 5'-UTR
18℃ ΔG = -13.92

Figure S6

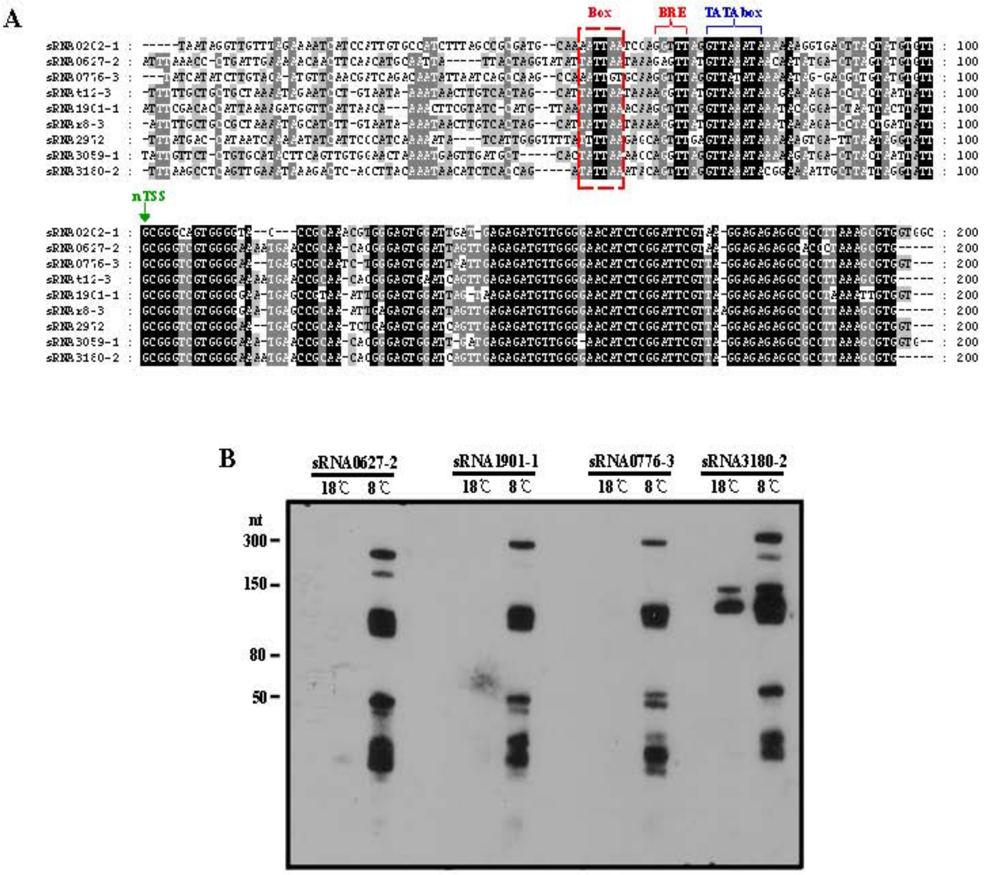
A
Box
BRE
TATA box
nTSS
B
sRNA0627-2
sRNA1901-1
sRNA0776-3
sRNA3180-2
18℃
8℃
nt
300
150
80
50

Figure S7

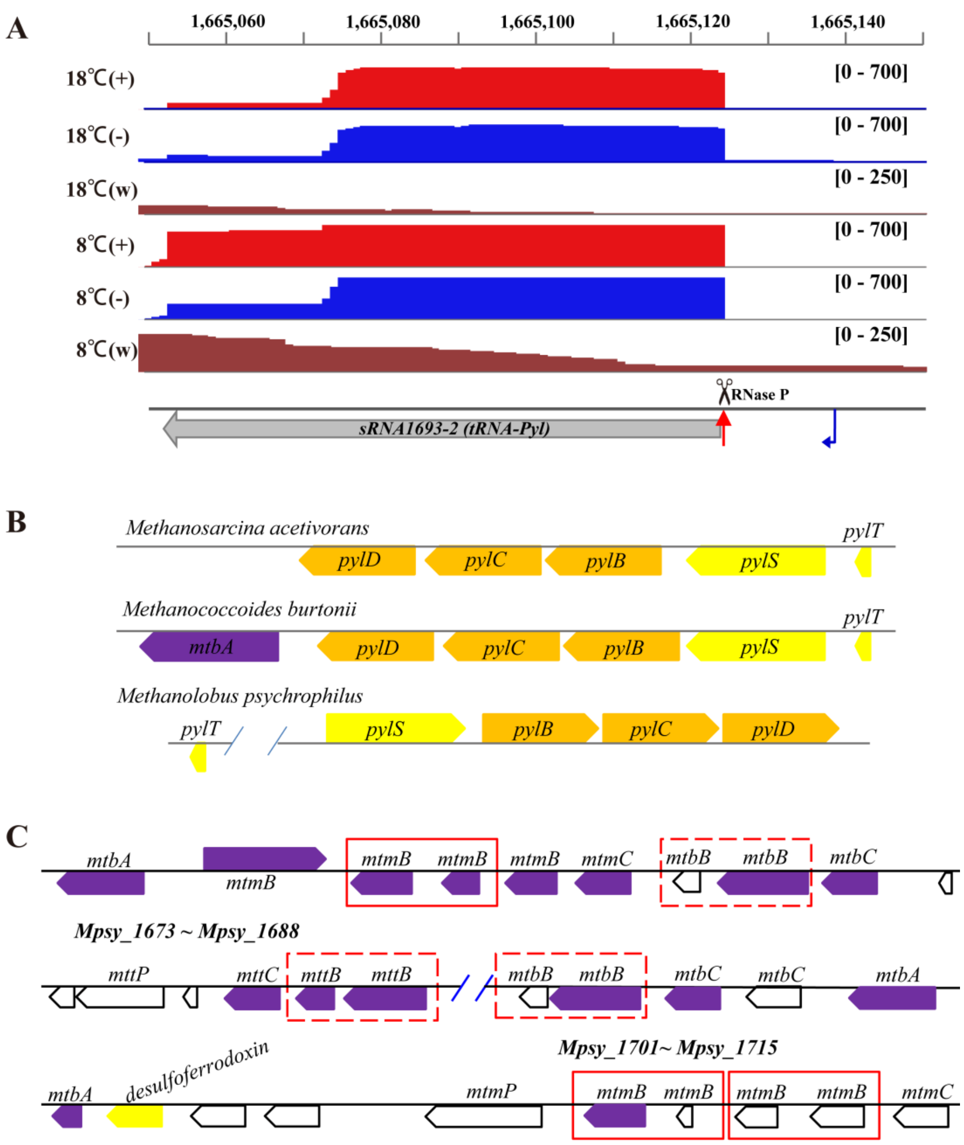
A
1,665,060
1,665,080
1,665,100
1,665,120
1,665,140
18℃(+)
18℃(-)
18℃(w)
8℃(+)
8℃(-)
8℃(w)
[0 - 700]
[0 - 700]
[0 - 250]
[0 - 700]
[0 - 700]
[0 - 250]
RNase P
sRNA1693-2 (tRNA-Pyl)
B
Methanosarcina acetivorans
pylT
pylD
pylC
pylB
pylS
Methanococcoides burtonii
pylT
mtbA
pylD
pylC
pylB
pylS
Methanolobus psychrophilus
pylT
pylS
pylB
pylC
pylD
C
mtbA
mtmB
mtmB
mtmB
mtmB
mtmC
mtbB
mtbB
mtbC
Mpsy_1673 ~ Mpsy_1688
mttP
mttC
mttB
mttB
mtbB
mtbB
mtbC
mtbC
mtbA
Mpsy_1701~ Mpsy_1715
mtbA
desulfoferrodoxin
mtmP
mtmB
mtmB
mtmB
mtmB
mtmC

Figure S8

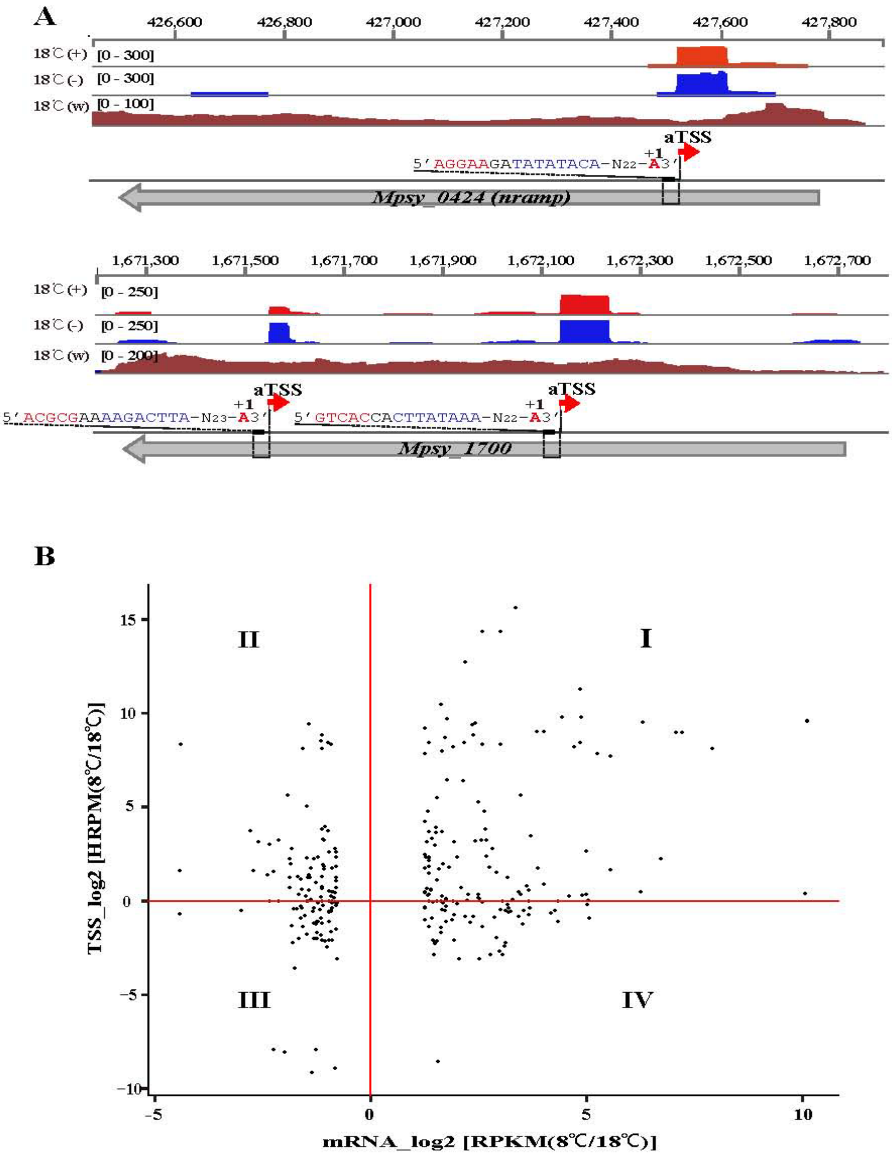
A
426,600
426,800
427,000
427,200
427,400
427,600
427,800
18℃(+) [0 - 300]
18℃(-) [0 - 300]
18℃(w) [0 - 100]
aTSS
+1
5′AGGAAGATATATACA-N22-A3′
Mpsy_0424 (nramp)
1,671,300
1,671,500
1,671,700
1,671,900
1,672,100
1,672,300
1,672,500
1,672,700
18℃(+) [0 - 250]
18℃(-) [0 - 250]
18℃(w) [0 - 200]
aTSS
+1
5′ACGCGAAAAGACTTA-N23-A3′
aTSS
+1
5′GTCACCACTTATAAA-N22-A3′
Mpsy_1700
B
II
I
III
IV
TSS_log2 [HRPM(8℃/18℃)]
mRNA_log2 [RPKM(8℃/18℃)]
15
10
5
0
−5
−10
−5
0
5
10

Figure S9

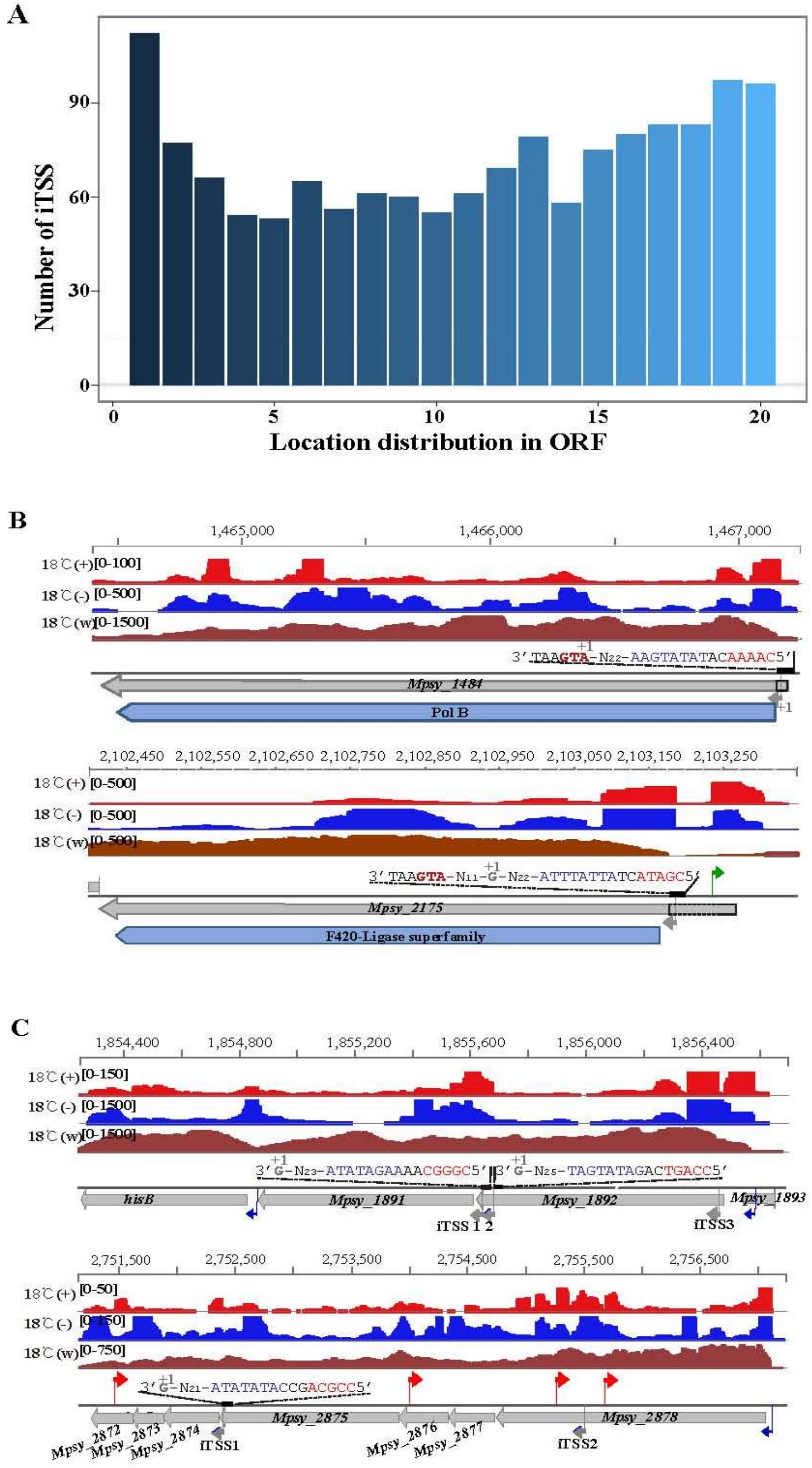
A
Number of iTSS
Location distribution in ORF
B
Mpsy_1484
Pol B
Mpsy_2175
F420-Ligase superfamily
C
hisB
Mpsy_1891
Mpsy_1892
Mpsy_1893
iTSS3
Mpsy_2875
Mpsy_2876
Mpsy_2877
Mpsy_2878
iTSS1
iTSS2

Figure S10

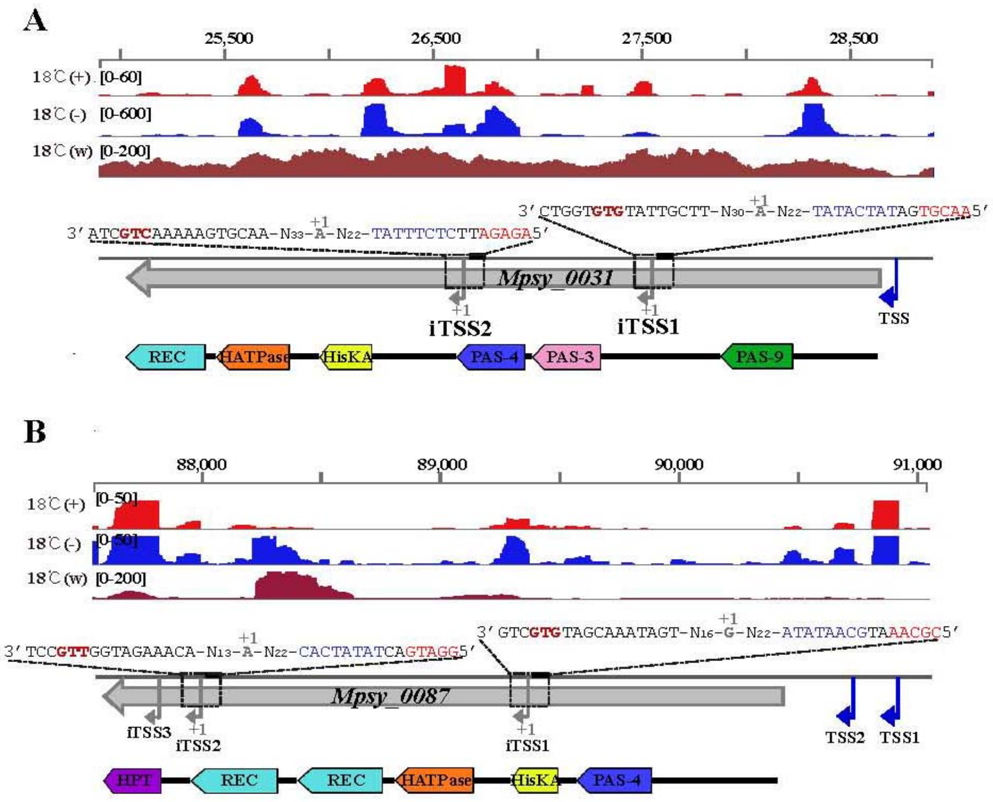
A
25,500
26,500
27,500
28,500
18℃(+) [0-60]
18℃(-) [0-600]
18℃(w) [0-200]
3′ATCGTCAAAAAGTGCAA-N33-A-N22-TATTTCTCTTAGAGA5′
3′CTGGTGTGTATTGCTT-N30-A-N22-TATACTATAGTGCAA5′
+1
Mpsy_0031
iTSS2
iTSS1
TSS
REC
HATPase
HisKA
PAS-4
PAS-3
PAS-9
B
88,000
89,000
90,000
91,000
18℃(+) [0-50]
18℃(-) [0-50]
18℃(w) [0-200]
3′TCCGTTGGTAGAAACA-N13-A-N22-CACTATATCAGTAGG5′
3′GTCGTGTAGCAAATAGT-N16-G-N22-ATATAACGTAAACGC5′
Mpsy_0087
iTSS3
iTSS2
iTSS1
TSS2
TSS1
HPT
REC
REC
HATPase
HisKA
PAS-4

Figure S11

Supplementary Tables

Table S1. Overview of the sequencing data information

Culture temperature	Library type	rRNA (%)	Clean data#	Mapping rate (%)	Mapped reads
18°C	(+)	12.27	11,555,975	52.72	6,092,170
	(−)	17.20	28,610,456	75.09	21,482,367
	(w)	1.24	32,655,016	98.58	32,190,288
8°C	(+)	2.45	13,080,637	33.37	4,364,495
	(−)	11.24	17,228,189	55.50	9,561,136
	(w)	1.47	32,441,532	98.21	31,859,599

#, clean data shows the number of total sequenced reads with rRNA removed.

Table S2. Primers and probes used in this study

Primer	Sequence (5′→3′)	Application
5′-RACE-F	CAGACTGGATCCGTCGTC	5′-RACE
0075-5RACE-RT	TCCCCTCAAACCCAAAGATCG	5′-RACE
0075-5RACE-R	GGCCTGTCAGCGTCGTCAC	5′-RACE
0406-5RACE-RT	GCGATCGTCTGGCGGTACTCT	5′-RACE
0406-5RACE-R	CCTGGACTATCGATTGCACATT	5′-RACE
0869-5RACE-RT	CACCCGGACGCTGCGAGAT	5′-RACE
0869-5RACE-R	CATCCATGTCTACACCGAACCTT	5′-RACE
1146-5RACE-RT	CCGGCCACGAATTAAACCTC	5′-RACE
1146-5RACE-R	CCTCGTCTTGGTCTGTGTCCT	5′-RACE
1554-5RACE-RT	CCCATGCTATCCAGTCTCCGTAA	5′-RACE
1554-5RACE-R	GCGTGCTTCCGTCCCCTC	5′-RACE
1637-5RACE-RT	CCGAAGGTGACACTCCCAGATAG	5′-RACE
1637-5RACE-R	TGGGCAATCTCGAAGTTTAAC	5′-RACE
2938-5RACE-RT	AGGCCAAGCTTTTCCATCTGC	5′-RACE
2938-5RACE-R	GCGGATTTTGTAGGCCTGTTC	5′-RACE
3066-5RACE-RT	TTCCGTCGCCTTCTCTTGC	5′-RACE
3066-5RACE-R	GCGATGTCTTCAATTGTCACTTCA	5′-RACE
0075-PE-RT	TCCCCTCAAACCCAAAGATCG	Primer extension
0075-PE-F	TGTCATTGGAACTGAACATG	Primer extension
0075-PE-R	ACATGCTCCATCGGAAAGCC	Primer extension
0869-PE-RT	TTCAAAAAAACTTCGCCTCTT	Primer extension
0869-PE-F	CGATCAAAGCAAAATAGTTAAGA	Primer extension
0869-PE-R	CACTGAAGTCCTCATCATCCAG	Primer extension
5SDNAProbe	CGGACTTATCTTCTGTGTTCGGAAAGGGTA<br>CAGGAATTGCCCCGCCGCTATGGCCGCCA	Northern blot
C/Dbox1 DNA Probe	TGTGCCTCAGTACTCATAGGGTTCATCATCG<br>ATCAGAACGATTTTCTCATCACAGGCAC	Northern blot
C/Dbox2 DNA Probe	TAGCGCGGCATCAGTTAGAGTAATCATCATCCT<br>TTGCTCAGTGGGGGTAACTATCATCATCGCCGC	Northern blot
nTSS393711DNAProbe	AACGAATCCGAAATATTCCCCAATATTTCTTC<br>CTGGACATTGCGAGTGACATTACCCGC	Northern blot
nTSS2892333DNAProbe	TTGGTCTGCATATATCTCAGAAAAGTGTCTC<br>AATGGACATTTCGCACCCCGTTCTCCCC	Northern blot
nTSS602251DNAProbe	CCCCAACATCTCTCAACTAATCCACTCCCGT<br>GTTGCGGTTCATTTTCCCCACGACCCGC	Northern blot
nTSS1863145DNAProbe	TTCCCCAACATCTCTTACTAATCCACTCCCAA<br>TTTACGGCTCATTCCCCCACGACCCGC	Northern blot
nTSS760266DNAProbe	TTCCCCAACATCTCTCAATTAATCCACTCCCA<br>GATTGCGGCTCATTCCCCACGACCCGC	Northern blot
nTSS3071986DNAProbe	ATCTCTCAACTGATCCACTCCCGTGTTGCGGT<br>TCATTTTCCCCACGACCCGCAATACCT	Northern blot

Table S10. Top 10 most abundant transcripts in TSS semi-quantitative analysis of R15 cultured at two temperatures

No.	RNA annotation	18°C HRPM	RNA annotation	8°C HRPM
1	SRP RNA	76514	sRNA3016-1	80615
2	sRNA3016-1	27649	SRP RNA	49042
3	Mpsy_t49 (tRNA-Gly)	18316	Mpsy_t49 (tRNA-Gly)	25387
4	sRNA2126-2	16477	Mpsy_t27 (tRNA-Asn)	14286
5	sRNA2068-1	14852	sRNA0273-3	14214
6	Mpsy_1673 (mtbA)	8320	sRNA2068-1	11610
7	Mpsy_t45 (tRNA-Leu)	7214	sRNA2871	11419
8	Mpsy_t3 (tRNA-Leu)	6626	Mpsy_t45 (tRNA-Leu)	10303
9	Mpsy_0908 (mtaC)	6089	sRNA1901-1	9662
10	Mpsy_t39 (tRNA-Met)	4799	sRNA2126-2	6717

SRP: signal recognition particle.
